# Calcifying aponeurotic fibroma around posterior tibialis tendon in an elderly patient with flatfoot: A case report

**DOI:** 10.1097/MD.0000000000026803

**Published:** 2021-07-30

**Authors:** Sung Hun Won, Jahyung Kim, Jaeho Cho, Dong-Il Chun, Kwonwoo Kim, Young Yi

**Affiliations:** a Department of Orthopaedic Surgery, Bone & Joint center, Soonchunhyang University Seoul Hospital, Seoul, Republic of Korea; b Department of Orthopaedic Surgery, Seoul Hospital, Soonchunhyang University Seoul Hospital, Seoul, Republic of Korea; c Department of Orthopaedic Surgery, Chuncheon Sacred Heart Hospital, Hallym University, Chuncheon, Republic of Korea; d Department of Health Administration, Sejong Public Health Center, Sejong, Korea; e Department of Orthopaedic Surgery, Seoul Paik Hospital, Inje University, Seoul, Republic of Korea.

**Keywords:** calcifying aponeurotic fibroma, flat foot, posterior tibialis tendon

## Abstract

**Rationale:**

Calcifying aponeurotic fibroma (CAF) is a rare benign fibroblastic tumor that is commonly in the hand or foot of children or adolescents.

**Patient concerns:**

A 74-year-old female presented with a progressive pain on the medial foot for 3 years ago. The pain aggravated while walking or in a standing position for more than 20 minutes. She also complained of skin contact along the medial aspect of the foot while trying to wear a shoe.

**Diagnosis:**

Physical examination revealed a firm, immobile, nontender mass accompanied with flexible flatfoot. On the single heel raise test, loss of the balance and intensification of the pain were observed. Faintly calcified soft tissue mass is shown in plain radiographs without bone involvement. Magnetic resonance imaging revealed a subcutaneous mass with ill-defined circumscribed subcutaneous mass adherent to the thickened PTT.

**Interventions:**

The patient underwent a complete excisional biopsy, followed by medial displacement calcaneal osteotomy.

**Outcomes:**

The excised mass was diagnosed to be CAF on the histologic examination. At the 1-year follow-up, patient remained asymptomatic with no evidence of recurrence and all the radiographic parameters demonstrating flat foot improved.

**Lessons:**

This is the first case of CAF located at PTT presenting with both foot pain and functional disability. In this case, complete excision of the causative structure along with alignment correction can contribute to successful postoperative outcome.

## 1 Introduction

Calcifying aponeurotic fibroma (CAF), also known as juvenile aponeurotic fibroma, is an unusual, benign soft tissue neoplasm that generally occurs in the palm of the hand and sole of the feet.^[1]^ It usually presents in children and adolescents, with male predominance.^[2]^ This rare tumor classically presents as a painless, slow-growing firm tumor, with the tendency to infiltrate the surrounding soft tissue. However, in some cases, CAF may cause pain, discomfort, or limitation of movement.^[3]^ Among many of the radiographic examinations, Magnetic resonance imaging is known to be ideal diagnostic examination presenting heterogenous high and low signal intensity combination on T2 weighed images and intermediate signal intensity on T1 weighted images.^[4]^ Once characterized histologically, surgical excision is recommended.^[1]^

Posterior tibialis tendon (PTT), is the largest and most anterior located tendons within the medial ankle. It inserts broadly into the plantar medial mid foot and acts as powerful plantar flexor of the ankle, inverter of the mid foot, and a primary dynamic stabilizer of the medial longitudinal arch.^[5]^ As a result, PTT dysfunction may lead to adult acquired flatfoot deformity, ending up with medial longitudinal arch collapse.^[6]^ Here we report a case of an elderly patient who presented CAF at PTT and was successfully treated through surgical procedure.

## 2 Case report

This case report was approved by the Institutional Review Board of Inje University Seoul Paik Hospital (IRB No. 2021-01-005). The patient gave written informed consent for publication of this report and the accompanying images.

### 2.1 Preoperative evaluation

A 74-year-old female presented with a progressive pain on the medial foot for 3 years ago. The pain aggravated while walking or in a standing position for more than 20 minutes. In addition, she also complained of skin contact along the medial aspect of the foot while trying to wear a shoe. She was conservatively treated with painkillers and injections but the pain worsened and was recently combined with diffuse swelling along the foot. No medical history, including systemic inflammatory disease or neuromuscular deficiency, previous trauma, or surgical intervention was reported. On physical examination, she had flexible planovalgus with inversion of the inner arch visually observed during weight-bearing. Although no remarkable callosity was noticed and there was a 2 cm sized firm, immobile mass-like lesion without tenderness palpated along the medial aspect of the foot. On the single heel raise test, loss of the balance and intensification of the pain were observed.

No specific findings were observed on the patient's hematological examination.

**Figure 1:**
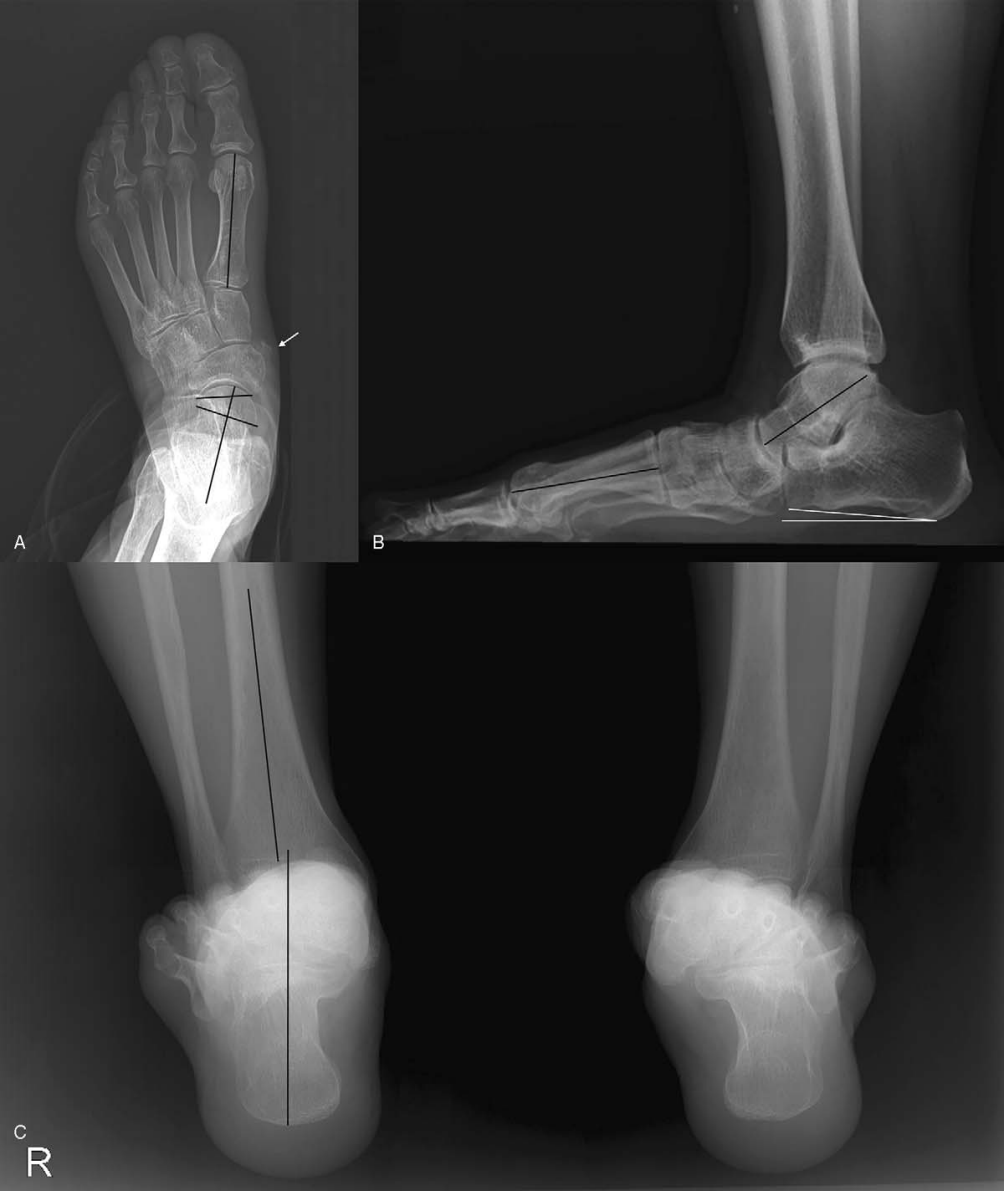
Preoperative weight-bearing foot anteroposterior (A) and lateral (B), and Saltzman hindfoot (C) view of the plain radiographs. (A) A faint, irregular-margin lesion with dense calcification is located medial to navicular bone without cortical erosion (arrow). Meary's is 9.5° and talonavicular uncoverage is 25%. (B) Lateral Meary's angle is 29° and calcaneal pitch angle is 6.3°. (C) Hindfoot alignment angle is −12.3° and hindfoot moment arm is −24.3 mm.

The plain radiographs of weight-bearing foot anteroposterior view showed a faint, irregular-margin lesion with dense calcification located medial to the navicular bone without cortical erosion. Anteroposterior Meary's angle was 9.5° and increased talonavicular uncoverage of 25% was observed. Weight-bearing lateral view showed decreased Meary's angle of 29° and calcaneal pitch angle of 6.3°. Saltzman hindfoot view showed valgus deformity with hindfoot alignment angle of −12.3° and hindfoot moment arm of −24.5 mm (Fig. 1).^[7]^

**Figure 2:**
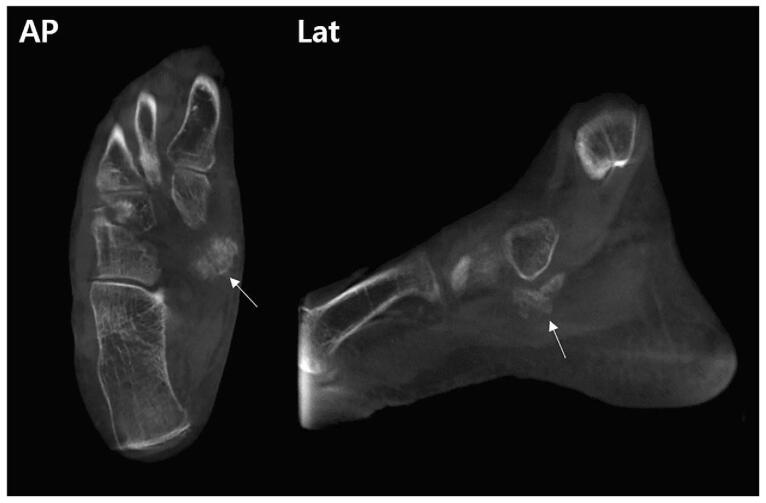
Preoperative CT images showing a 2 × 2 × 1 cm sized irregular ill-defined mass (arrow) placed inferomedial to navicular bone without demonstrating the corticomedullary continuity with the adjacent bone. CT = computed tomography.

**Figure 3:**
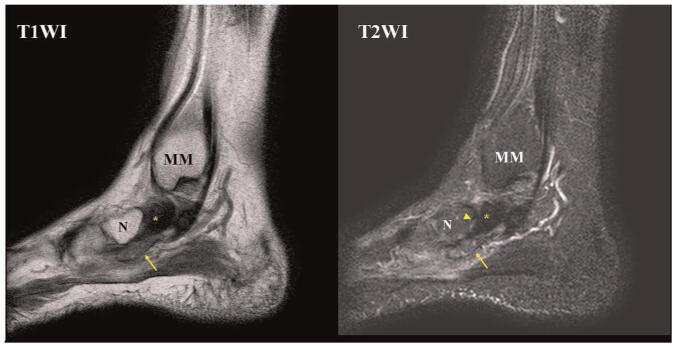
Preoperative MRI scans showing a low T1-weighted and heterogenous high T2-weighted signal intensity mass with ill-defined margins (arrow) at the navicular insertion of the PTT. PTT (asterisk) was thickened around its navicular insertion with high T2-weighed signal intensity, demonstrating a tendinosis (arrowhead). MM = medial malleolus, MRI = magnetic resonance imaging, N = navicular bone, PTT = posterior tibialis tendon.

To clarify the lesion, a computed tomography was performed, which showed a 2 × 2 × 1 cm sized irregular ill-defined mass placed inferomedial to navicular bone without demonstrating corticomedullary continuity with the adjacent bone (Fig. 2). Magnetic resonance image scans demonstrated a low T1-weighted and heterogenous high T2-weighted signal intensity mass with ill-defined margins at the navicular insertion of the PTT. Cortical integrity of the adjacent bones was maintained. In addition, PTT was thickened around its navicular insertion with high T2-weighed signal intensity, demonstrating a tendinosis (Fig. 3). The American Orthopedic Foot and Ankle Society ankle-hindfoot score was 52 points.^[8]^

### 2.2 Surgical procedure

**Figure 4:**
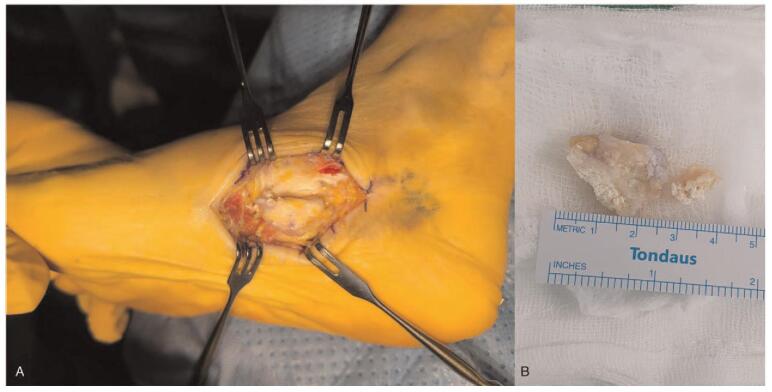
Intraoperative photographs. (A) 2 × 2 × 1 cm sized mass (asterisk) is noted to be circumscribed and adherent to the navicular insertion of the swollen, degenerated PTT (arrow) (B) Soft tissue mass accompanied with calcification that is fully excised from the PTT. PTT = posterior tibialis tendon.

An excisional biopsy and surgical exploration were performed under spinal anesthesia with tourniquet control. Intraoperatively, 2 × 2 × 1 cm sized mass with calcification was noted to be circumscribed and adherent to the navicular insertion of the swollen, degenerated PTT (Fig. 4). The mass was completely excised and sent to the pathology department for histologic examination. In order to realign the hindfoot valgus and relieve tension around PTT insertion, medial displacement calcaneal osteotomy (MDCO) using 6.5 mm partially threaded cannulated screw was performed additionally.

### 2.3 Postoperative progression

**Figure 5:**
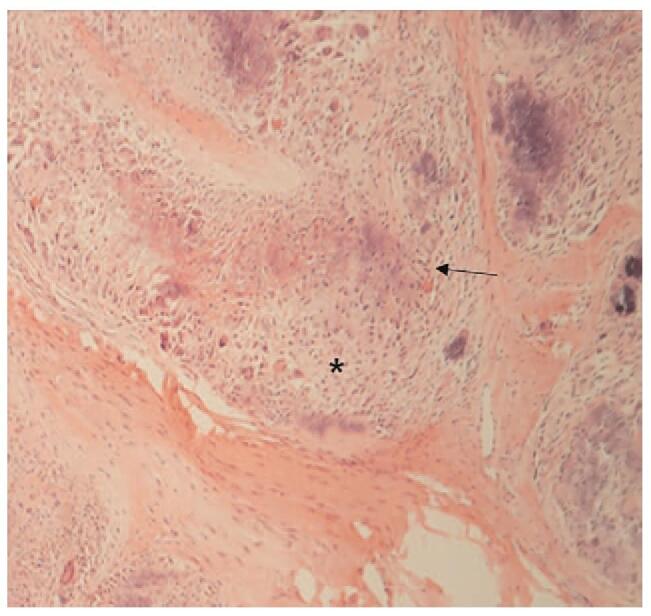
Microscopic appearance of CAF displays focal chondroid differentiation (arrow) with central calcification (asterisk), surrounded by spindle cell proliferation (Haematoxylin and eosin (H-E), ×100). CAF = calcifying aponeurotic fibroma, H-E = hematoxylin and eosin.

**Figure 6:**
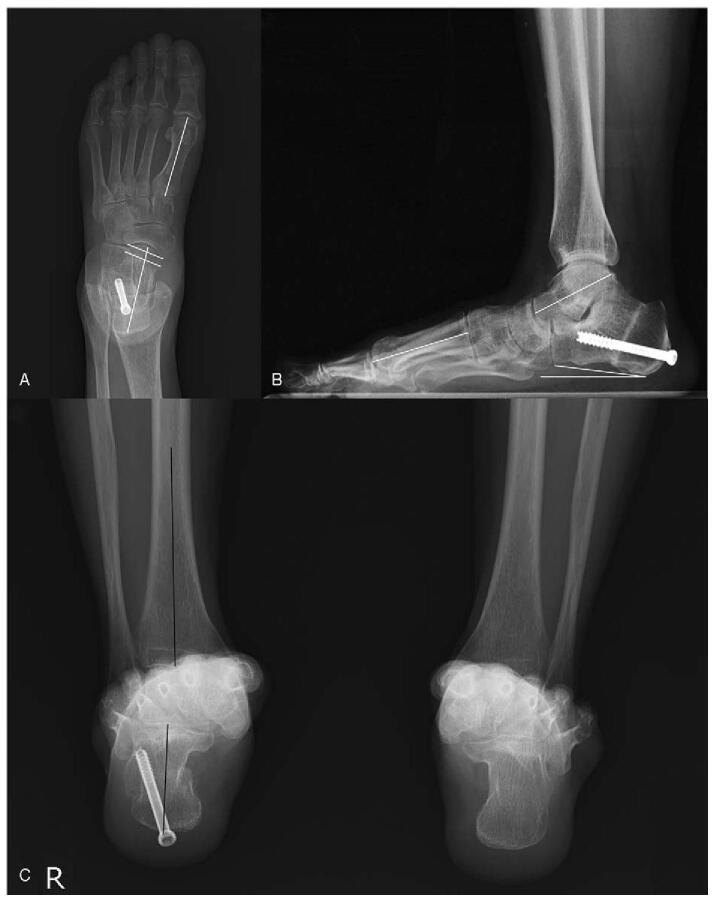
Postoperative 1-yr weight-bearing foot anteroposterior (AP) (A) and lateral (B), and Saltzman hindfoot (C) view of the plain radiographs. (A) There is no evidence of the newly appeared mass-like lesion. The AP Meary's angle is 1° and talonavicular uncoverage is 8%. (B) The osteotomized site of the calcaneus is completely fused. The lateral Meary's angle is −12° and calcaneal pitch angle is 7.3°. (C) Hindfoot alignment angle is −1.1° and hindfoot moment arm is −3.2 mm. AP = anteroposterior.

The histologic sample was confirmed to be CAF (Fig. 5). A short leg cast was applied for protection and the patient was allowed to fully bear weight and return to the regular activity 1 month after surgery. At the 1-year follow-up, patient remained asymptomatic, with no evidence of recurrence. The American orthopedic foot and ankle society ankle-hindfoot score improved to 92 points. In terms of radiographic examination, the osteotomized site of the calcaneus was completely fused and the radiographic parameters improved compared with those measured preoperatively: AP Meary's angle from 9.5° to 1°, talonavicular uncoverage from 25% to 8%, lateral Meary's angle from −29° to −12°, calcaneal pitch from 6.3° to 7.3°, hindfoot alignment angle from −12.3° to −1.1°, and hindfoot moment arm from −24.5 to −3.2 mm (Fig. 6).

## 3 Discussion

CAF is a rare benign fibrous tumor that commonly involves the dense fibrous connective tissue, that is deep fascia, tendons, or aponeuroses.^[9]^ Since first reported by Keasbey in 1953, variety of cases of CAF have been reported in the literature and these predominantly occur in the distal upper extremities.^[10,11]^ In succession to the hands, CAF is also known to involve the foot, although less frequently. Examples of the affected sites within the foot that are documented in the literature involve plantar and dorsal aspect of the foot, hallux, and Achilles tendon.^[1,4,10,12]^ To best of our knowledge, this is the first reported case of CAF that occurred at PTT. Besides, due to the significant impact of PTT within the foot while at stance or at gait, functional deterioration was also manifested in this case.

Because CAF is clinically presented as an ill-defined lesion, its recurrence rate is known to be as high as 50% after surgery.^[10]^ As a result, the recommended treatment for CAF is known to be complete excision after incisional biopsy results are confirmed. In this study, however, the mass was located at the PTT, the PTT turned out to be swollen intraoperatively. In addition, flexible flatfoot deformity accompanied with valgus hindfoot alignment was also present. Although whether CAF primarily caused PTT dysfunction is unclear, it seems to be reasonable to imply that iatrogenic mass-effect of the mass and hindfoot valgus deformity together aggravated the increased PTT tension, consequently bringing out out both medial foot pain and diminished PTT function. Therefore, authors thought that surgical excision of the mass alone was not enough to thoroughly fulfill the patient concerns.

Aside from its role in plantar flexion of the ankle joint and adduction and supination of the subtalar joint, PTT also helps support the foot arches.^[5]^ As a result, dysfunction of the PTT may result in abnormal foot biomechanics, eventually ending up with the acquired flatfoot deformity.^[13]^ In this study, PTT was elongated and swollen, accompanied with flexible flatfoot. In addition, single-leg raise test was not possible, because of the weakened PTT. Based on the classification for posterior tibialis tendon disease (PTTD) suggested by Bluman et al, the patient condition presented in this case corresponds with stage 2.^[14]^ In terms of treatment modality for stage 2 PTTD, surgical approach is usually recommended if the pain persists despite conservative treatments including wearing the orthoses.

Intraoperatively, MDCO was additionally performed along with complete excision of the mass. By medially shifting the calcaneal tubercle, MDCO can not only improve the deformity, but also effectively relieve the tension around the PTT attachment.^[15,16]^ Numerous studies have reported significant pain relief, corrected alignment, and improved function following MDCO.^[17–19]^ As a result, MDCO is generally accepted for mild flat foot deformity.^[15]^ In addition, Schon et al suggested isolated MDCO when isolated hindfoot valgus exists with adequate talonavicular joint coverage (less than 35% to 40% uncoverage) and a lack of significant forefoot supination, varus, or abduction.^[20]^ Since adequate talonavciular joint coverage was detected in this case with talonavicular joint uncoverage of 25%, only the MDCO was performed and satisfactory postoperative outcome could be achieved.

## 4 Conclusion

In conclusion, we encountered the first case of CAF located at PTT presenting with both foot pain and functional disability. In this case, complete excision of the causative structure along with alignment correction can contribute to successful postoperative outcome.

## Author contributions

**Conceptualization:** Jaeho Cho, Young Yi.

**Funding acquisition:** Sung Hun Won, Young Yi.

**Investigation:** Young Yi.

**Resources:** Kwonwoo Kim.

**Validation:** Dong-Il Chun.

**Writing – original draft:** Sung Hun Won.

**Writing – review & editing:** Jahyung Kim.
